# Congenital cataract: An ocular manifestation of classical homocystinuria

**DOI:** 10.1002/mgg3.1742

**Published:** 2021-08-02

**Authors:** Neelam Saba, Saba Irshad

**Affiliations:** ^1^ School of Biochemistry and Biotechnology University of the Punjab Quaid‐e‐Azam Campus Lahore Pakistan

**Keywords:** congenital cataract, homocystinuria, metabolic, missense variant

## Abstract

**Background:**

Homocystinuria is an autosomal recessive metabolic disorder occurring due to the defects in cystathionine‐β‐synthase enzyme. The study was carried out to investigate a Pakistani family presenting bilateral congenital cataract with symptoms of classical homocystinuria at LRBT Free Eye Hospital, Lahore, Pakistan.

**Methods:**

Three affected individuals of the family presented skeletal deformations, intellectual disability, speech delay, and myopia with bilateral congenital cataract. Genetic analysis on DNA samples from affected individuals was done through whole exome sequencing to identify underlying genetic variant causing disease phenotypes in the family. In silico analysis was done to predict the effect of variation on the structure of mutant protein.

**Results:**

A missense allelic variant (NM_000071.3: c.253G>A) of the *CBS* gene was revealed which may affect the catalytic activity of the substituted (NP_000062.1: p.G85R) protein by disrupting the folding of the enzymatic protein. High levels of homocysteine were observed in the plasma of affected individuals. This is the first report of this genetic variant from Pakistan causing homocystinuria and congenital cataract in association.

**Conclusion:**

This variant was reported first time in association with congenital cataract instead of ectopia lentis. Congenital cataract was developed secondarily in these patients and provided a clue for the early diagnosis of metabolic disorders like homocystinuria to prevent further complications and morbidity.

## Graphical abstract

This study was done to identify the genetic cause of CC in a Pakistani family. Through WES, a missense SNP of the CBS gene responsible for classical homocystinuria was revealed, which developed CC secondarily in the patients.

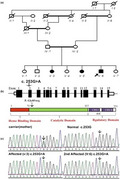

## 1 INTRODUCTION

Homocystinuria (MIM# 236200) is the autosomal recessive metabolic genetic disorder due to the deficiency of cystathionine‐β‐synthase (CBS) enzyme (Mudd et al., 1995). CBS enzyme (EC#4.2.1.22) catalyses the conversion of homocysteine and serine to cystathionine by transfer of sulfur in homocysteine metabolism pathway to form cysteine (Selhub, 1999). The CBS enzyme in humans is encoded by the *CBS* gene (MIM#613381) (NG_008938.1), which has been mapped on chromosomal location 21q22.3 with 17 exons. The enzymatic protein with catalytic activity is a homotetrameric protein with four subunits; each subunit is made of 551 amino acids (NP_000062.1). To date, 198 *CBS* allelic variants have been reported for classical homocystinuria (HGMD Professional ^®^ 2020.3 total; The Human Gene Mutation Database, 2019).

Classical homocystinuria results in higher levels of homocysteine (Ref. 15 µM) and methionine (Ref. 14–64 µM/L) and lower levels of cystathionine and cysteine in blood and urine (Saudubray et al., 2016). Homocystinuria presents with a multitude of symptoms mainly related to ocular, nervous, skeletal and vascular systems of the body. The affected individuals are healthy at the time of birth but they show developmental delay during first years of life. The reported global incidence of homocystinuria is estimated to be 1 in 200,000 (Al‐Sadeq & Nasrallah, 2020).

In the eye the most commonly described manifestations of homocystinuria are ectopia lentis and myopia. Other ocular defects which have been reported are spherophakia, aniridia, retinal cysts, retinal detachment and optic atrophy (Presley & Sidbury, 1967).

Studies have reported that long‐term exposure of human and animal cells to higher concentrations of homocysteine results in the unfolded protein responsible for congenital cataract formation (Tan et al., 2015). In underdeveloped countries like Pakistan, these types of metabolic disorders remain undiagnosed due to lack of screening tests until later age.

Here we report a bilateral congenital cataract‐affected Pakistani family with symptoms of classical homocystinuria. The affected individuals experienced the symptoms of the disease since early childhood.

## 2 MATERIALS AND METHODS

### 2.1 Ethical compliance

This study was approved by the Ethical Committee of the University of the Punjab and Institutional Review Board of School of Biochemistry and Biotechnology (SBB), University of the Punjab, Lahore Pakistan, and performed according to the Declaration of Helsinki.

### 2.2 Family ascertainment and DNA specimens

An affected family with three affected individuals (V: 3, V: 5 and V: 6) presenting with symptoms of classical homocystinuria and bilateral congenital cataract was ascertained from LRBT (Layton Rehmatullah Benovelent Trust) free eye hospital, Lahore Pakistan. Affected family members underwent slit lamp examination and visual acuity tests with dilated pupils in the hospital by expert ophthalmologists. Comprehensive family history was obtained from the elder family member and photographs of the affected individuals were taken. Final pedigree was constructed with CYRILLIC software. Written informed consent was obtained from parents of the affected individuals.

Five millilitre venous blood samples were collected from three affected and five unaffected family members in K3EDTA vacutainers (KANTEX). All the vacutainers were labelled and kept at 4°C until DNA extraction. Salting out procedure was used for extracting DNA from the whole blood (Helms, 2002).

### 2.3 Whole exome sequencing

DNA sample of one affected individual (V: 6) was subjected to whole exome sequencing (WES). WES was performed using Sure Select Human All Exon Kit v6 for Exom Capture (Agilent Technologies, USA) and Illumina HiSeq 4000 platform for sequencing. A pipeline of published algorithms including Burrows‐Wheeler aligner tool (BWA), SAMtools, PICARD, and the Genome Analysis Toolkit (GATK) was used for exome data analysis in a sequential manner. Human genome build 37 (UCSC hg19) was used as a reference genome to align sequence reads. Variant filtering was done as described in previous studies (Ansar et al., 2018; Riazuddin et al., 2020).

Exome data were filtered for allelic variants of the genes related to disease phenotypes such as congenital cataract, intellectual disability, skeletal deformations, and speech delay in the affected individuals. Variants were selected with a minor allele frequency of <0.02 in Exome Aggregation Consortium (ExAC) (http://exac.broadinstitute.org).

### 2.4 Analysis of co‐segregation

An allelic variant of the *CBS* gene (NM_000071.3: c.253G>A) as a possible cause of associated clinical phenotypes in the affected family was validated for co‐segregation by Sanger sequencing.

Sanger sequencing primers (forward primer: 5′CAGGGCAAAATCTCCAAAAA3′, reverse primer: 5′GCCCAGTGTAGATGGAGGAA3′) were designed for exon, having candidate variant using Primer 3 software for primer synthesis (https://bioinfo.ut.ee/primer3‐0.4.0/primer3/). Amplification reactions were performed with 40 ng/µl of DNA using Taq DNA polymerase in 25 µl reactions (Table S1 and Figure S1). Sequencing was performed using a Big Dye^®^ terminator v3.1 Cycle Sequencing Kit (Applied Biosystems, USA) with ABI PRISM^®^ 3730 genetic analyser. After sequencing, data were analysed with SeqMan program of Lasergene (DNASTAR).

### 2.5 In silico analysis

The pathogenicity scores of the variant were predicted through SIFT, PolyPhen‐2 and CAAD. The pathogenicity of the reported variant in the *CBS* gene (NM_00071.3: c.253G>A) was predicted by structure modelling of mutant enzymatic protein (NP_000062.1: p.Gly85Arg) through HOPE (Have your Protein Explained) software (https://www.cmbi.umcn.nl/hope).

### 2.6 Biochemical analysis

After exome sequencing biochemical analysis was done to confirm the high levels of homocysteine in serum of the three confirmed cases of homocystinuria V: 3, V:5 and V:6. Reference value for normal range of homocysteine was considered <15 µM/L in serum.

## 3 RESULTS

### 3.1 Clinical findings

**FIGURE 1:**
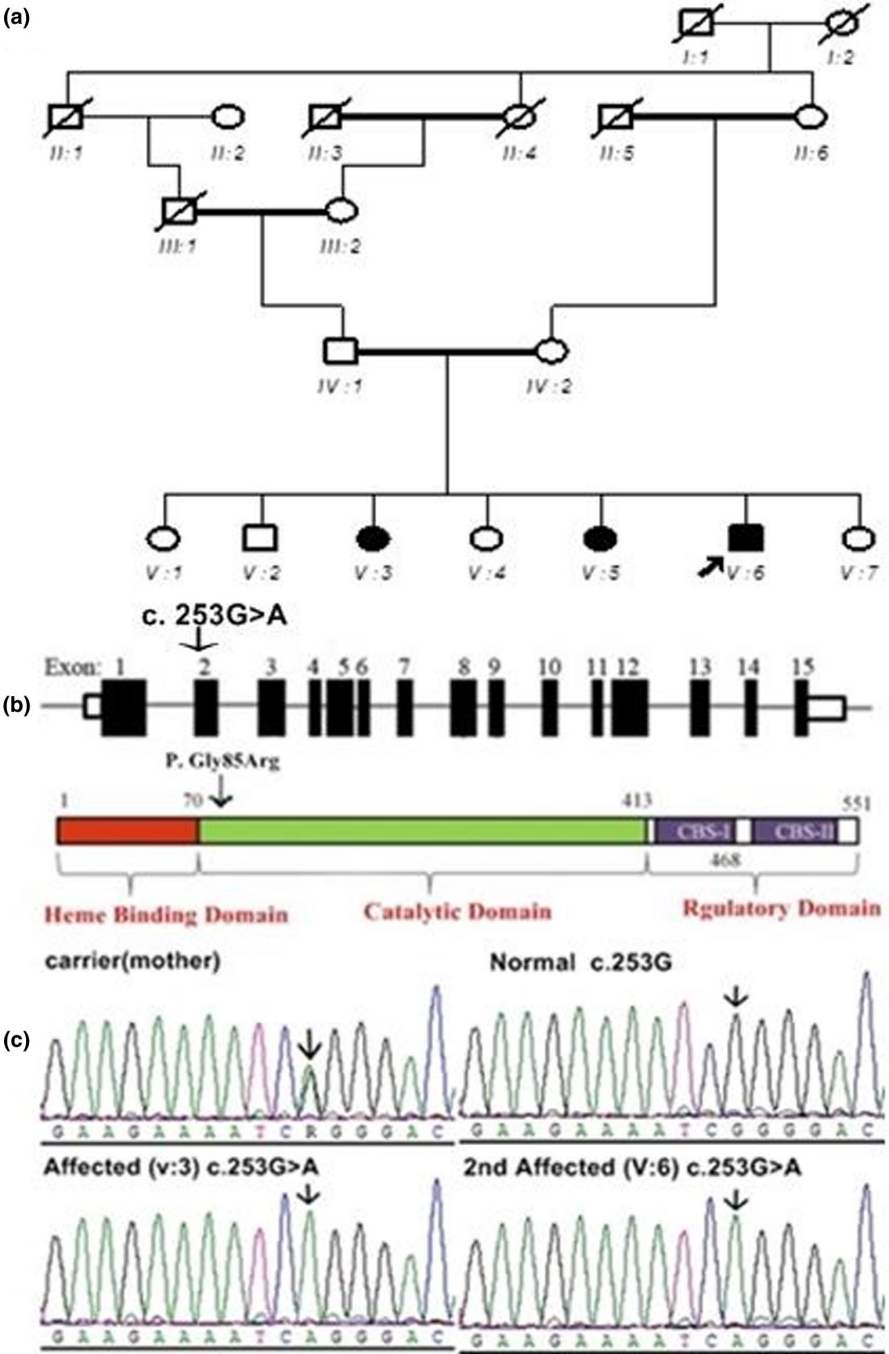
(a) Pedigree of the affected family with autosomal recessive mode of inheritance, arrowhead indicates the proband. (b) *CBS* gene cDNA and protein. Transcript showing 15 coding exons. Nucleotide change c.253G>A in coding exon 2 is indicated (noncoding exons are not shown here). The *CBS* gene codes for a 551 amino acid protein with three domains: heme binding domain, catalytic domain, and two regulatory domains CBS‐I and CBS‐II (UniProtKB‐P35520.1). (c) Sequence chromatograms of homozygous affected, heterozygous carrier, and normal individuals of the family showing segregation of the mutation

The affected family with three affected individuals was recruited from a rural area of Punjab province of Pakistan. Both parents were normal with second grade of consanguinity. Autosomal recessive pattern of inheritance was ascertained from the pedigree (Figure 1a). All affected individuals were reported to be affected since birth. Clinical data of affected individuals are given in Table 1. Photographs of affected individuals are given in Figure S2.

**TABLE 1:** Clinical phenotypes of affected individuals

Individual ID	Sex	Age at onset	Age at diagnosis	Visual acuity OD/OS	Clinical findings
V:3	Female	1 year	10 years	CF/CF	B/L cataract (OP), myopia, skeletal deformations
V:5	Female	1.5 years	7 years	CF/PL	Unilateral cataract, myopia, squint, skeletal deformations, intellectual disability, speech delay
V:6	Male	6 months	5 years	PL/PL	B/L cataract, skeletal deformation, retarded growth

_Abbreviations: B/L, bilateral; CF, counting fingers; OD, oculus dextrus (right eye); OP, operated; OS, oculus sinister (left eye); PL, perception of light_.

### 3.2 Whole exome analysis

After exome sequencing, data were filtered and analysed for causative variant of clinical phenotypes observed in patients. A missense variant (NM_000071.3: c.253G>A) was identified in the *CBS* gene responsible for symptoms of classical homocystinuria. The gene is located on chromosome 21 in the chromosomal location 21q22.3. This variant in exon 4 of the transcript (NM_001321073.2) substitutes an amino acid arginine for glycine at position 85th of the translated protein product (NP_000062.1: p.Gly85Arg) (Figure 1b). This variant of the *CBS* gene was reported in genomAD (https://gnomad.broadinstitute.org) db SNP, 1000 genomes databases but was not found in Exac database (http://exac.broadinstitute.org). The pathogenicity score predicated by SIFT (http://sift.jcvi.org), PolyPhen‐2 (http://genetics.bwh.harvard.edu/pph2) and CADD software in ClinVar (https://cadd.gs.washington.edu/) was found to be 0, 1 and 25, respectively showing deleterious effect of the mutation on protein function.

### 3.3 Segregation analysis

Sanger sequencing was done to confirm the autosomal recessive mode of inheritance of the *CBS* gene variant (NM_000071.3: c.253G>A) in the affected family. The results were compared with the reference sequence (NC_000021.9) in GenBank. All affected family members were homozygous for this variant while all carriers were heterozygous normal (Figure 1c).

### 3.4 In silico analysis

**FIGURE 2:**
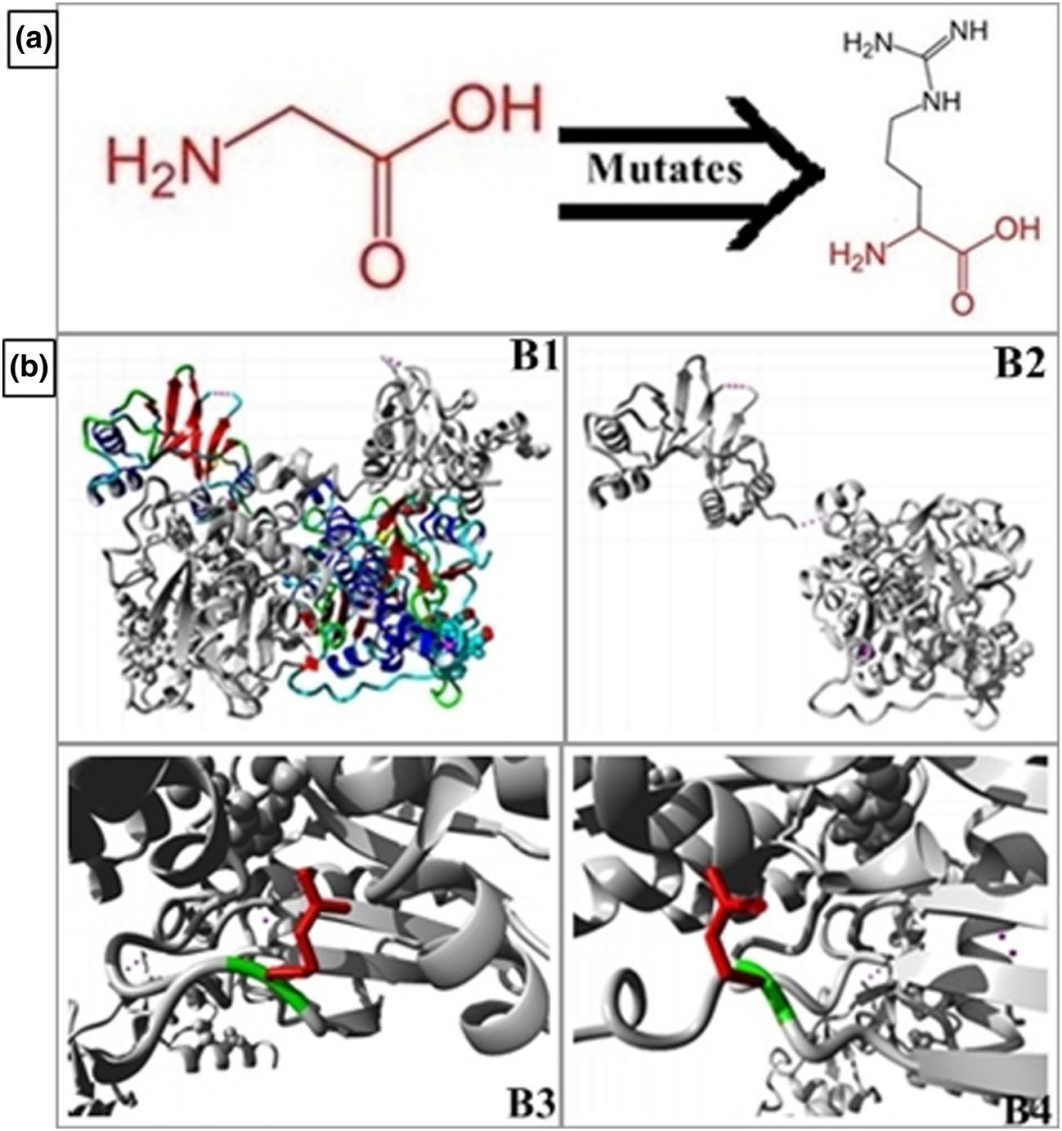
In silico pathogenicity prediction of CBS mutant p.G85R. (A) Structure of the wild‐type (left) glycine and mutant (right) arginine amino acid residue showing size and side chain differences (B) Structural prediction of pathogenic protein through HOPE software, structure of native CBS protein (B1), structure of mutant CBS protein (B2), B3 and B4 closer view of variant with wild‐type amino acid in green and mutant amino acid in red disrupting the interaction between domains and hence folding of the protein

HOPE software was used to predict the possible pathogenic effect of the mutation on the structure of the CBS protein (NP_000062.1: p.Gly85Arg). It was revealed that mutant amino acid arginine is larger in size and positively charged as compared to wild‐type glycine amino acid residue (Figure 2A) which is hydrophobic and smaller in size affecting the interaction between two adjacent peptide domains. Glycine is the most flexible of all the amino acids making torsion angles possible at this position (buried at the core of domain). The torsion angles made by mutant residue (arginine) are unusual due to larger size disturbing the structural conformation and hence folding of the mutant protein (Figure 2B).

### 3.5 Biochemical analysis

In affected individuals very high level of homocysteine (>100 µM) was observed in serum. The level of homocysteine in patients V3, V5 and V6 was 230 µM/L, 245 µM/L and 150 µM/L respectively.

## 4 DISCUSSION

In this study a homozygous missense change (NM_000071.3: c.253G>A; p.Gly85Arg) was identified through whole exome sequencing in the catalytic domain of the *CBS* gene causing classical homocystinuria in three affected siblings of a Pakistani consanguineous family. Initially, this family was diagnosed with bilateral congenital cataract and severe myopia manifesting the secondary ocular complications of homocystinuria. The reported mutation segregates in autosomal recessive mode in all affected individuals of the family. The *CBS* gene encodes a 63KD enzymatic protein which requires pyridoxal phosphate and heme as a cofactor for its transulfuration activity. The catalytic activity of the CBS protein (UniProtKB‐P35520.1) is present in N‐terminal 409 amino acid domain while C‐terminal regulatory domain is composed of 142 amino acids. It has been studied that most mutations that occur in regulatory domain do not affect the catalytic activity of the protein but interfere with the activation of the enzyme (Shan et al., 2001).

Homocystinuria is an autosomal recessive metabolic disorder which occurs when two affected copies of the gene are transmitted from two carrier parents, therefore in Asian countries incidence of metabolic disorders like homocystinuria is high due to consanguineous marriages.

The reported mutation (NM_000071.3: c.253G>A; p.Gly85Arg) was identified in the catalytic domain of the protein which is highly conserved and pathogenicity of this mutation is related to the catalytic activity of the protein. We have done in silico structural predictions of the mutant protein by HOPE software in this study which revealed possible pathogenic effects of this mutation by disturbing the three dimensional structure and folding of the protein domains. So these structural changes may affect the catalytic activity of the mutant protein. This variant in the *CBS* gene was first reported in the Danish population (Kraus et al., 1999) but no studies were done on the structure–function relationship of this variant. The two most frequently reported mutations in homocystinuria patients around the world are p.G307S (31%) and p.I278T (24%) (Kaur et al., 2020) associated with myopia and ectopia lentis and both of these are present in the catalytic domain affecting the catalytic activity of the protein.

In the Pakistani population, two novel mutations, a missense change (c.467T>C; p.L156P) and in frame deletion (c.808_810del; p.E270del), were reported in the catalytic region of the protein causing homocystinuria, ectopia lentis and subluxation of the eye lens in patients (Ibrahim et al., 2018). There are few reports on screening of this metabolic genetic disorder in Pakistani population. In 2018, a 7‐year‐old male child with bilateral congenital cataract was reported with homocystinuria (Kamat et al., 2018).

The reported mutation (p.G85R) in this study was identified previously in the *CBS* gene in Brazilian patients (Poloni et al., 2018) in association with dislocated lens, but no report of this mutation is available in any Asian population to date. In our study, association of this variant was observed with congenital cataract in three affected individuals. Recently, screening and biochemical analysis of homocystinuria patients with congenital cataract was done in Chennai, India (Sulochana et al., 2000). A significant association of classical homocystinuria was observed with congenital cataract in affected children under 10 years of age. In classical homocystinuria patients, low bioavailability of cysteine required for the synthesis of reduced glutathione reflect the possible association of congenital/developmental cataract in homocystinuria, as reduced cystathionine is required for antioxidative properties. The effect of this variant on catalytic activity of the CBS protein in yeast strain (*S*. *cerevisiae*) was also studied. Partial enzymatic activity of the mutant protein (p.G85R) was observed in the presence of vitamin B6. A significant decrease in the production of antioxidant glutathione was also observed in the mutant yeast suggesting the metabolic consequences of this variant (Mayfield et al., 2012).

Early detection of homocysteine in plasma/urine, methionine restricted diet, folic acid and vitamin B6 supplement is the best treatment for homocystinuria patients (Al‐Dewik et al., 2019). In future chemical chaperons to minimize the accumulation of misfolded protein and enzyme replacement therapy is being considered for homocystinuria treatment and clinical trials are being done on mice strain by administration of PEG‐CBS. The results showed the significant reduction in plasma homocysteine levels in CBS‐deficient mice (Al‐Sadeq & Nasrallah, 2020; Majtan et al., 2018).

## 5 CONCLUSION

A metabolic disorder like homocystinuria remains undetected until late age due to lack of screening tests in underdeveloped countries like Pakistan. In this congenital cataract‐affected family, cataract provided a clue for the diagnosis of homocystinuria; hence these types of metabolic disorders can be diagnosed by ocular manifestations. Early diagnosis of the metabolic disorders can help to minimize other systematic complications in patients by providing appropriate therapy.

A consanguineous marriage is the main factor for the inheritance of autosomal recessive disorders. As homocystinuria is an inherited metabolic disorder, it cannot be cured; however, complications can be prevented by a methionine‐restricted diet. It has been suggested that vitamin B12 and folic acid are involved in homocysteine metabolism and can lower homocysteine in serum; therefore, future studies should be targeted toward the response of patients to these therapies.

## CONFLICTS OF INTEREST

The authors have no conflicts of interest to declare.

## AUTHOR CONTRIBUTIONS

Saba Irshad designed the study. Neelam Saba acquired and analysed the data and made a final draft of the study.

## ETHICAL APPROVAL

This study was approved by the ethical committee of the University of the Punjab, Pakistan and Institutional Review Board of the Institute of Biochemistry and Biotechnology, University of the Punjab.

## INFORMED CONSENT

After obtaining ethical approval from review committees of the University of the Punjab, Lahore Pakistan, for this study, written informed consent was obtained from parents of the affected individuals for blood sample collection.

## Supporting information

Table S1‐Fig S1‐S2

## Data Availability

Any supporting data for this study will be available on request. Phenotypes of the affected individuals supporting this study are obscured due to ethical restrictions and attached in the form of photographs in the supplementary data file with the article.
