# Supplementary material for: Congenital cataract: An ocular manifestation of classical homocystinuria
Source: Mol Genet Genomic Med. 2021 Aug 2;9(9):e1742. doi: 10.1002/mgg3.1742 (PMC8457696; doi:10.1002/mgg3.1742)
Supplement: Supplementary file 1 — Table S1‐Fig S1‐S2 [file MGG3-9-e1742-s001.docx]

Title: Congenital Cataract; an Ocular Manifestation of Classical Homocystinuria

Table S1: composition of 25uL PCR reaction

| Ingredients | Stock Conc. | Final Conc. | Volume |
| --- | --- | --- | --- |
| Genomic DNA | 40 ng | 80 ng | 2 µL |
| MgCl2 | 25 mM | 3 mM | 3 µL |
| Primer (Forward+Reverse) | 10 pM | 0.4 pM | 1 µL |
| DNTPs (dATP,dGTP,dCTP,dTTP) | 10 mM | 0.2mM | 0.5 µL |
| PCR Buffer | 10 X | 1 X | 2.5 µL |
| Taq Polymerase | 5U/ µL | 1U | 0.2 µL |
| dH2O |  |  | Add to make volume 25µL |


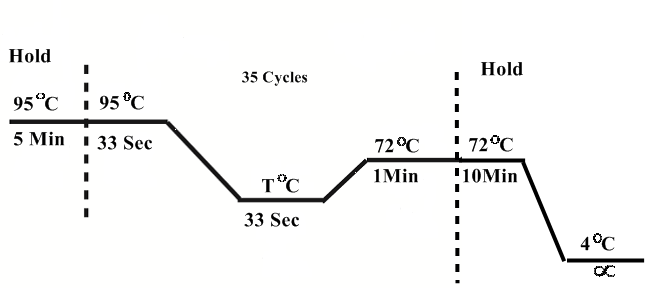


Figure S1: Thermocyler program. T°C (Anealing temperature optimized as 57-60 °C).


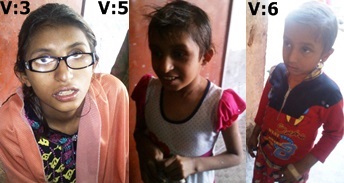


Figure S2: Photographs of the individuals showing phenotypes of Classical Homocystinuria (skeletal deformations, brown hair, and intellectual disability).
